# Thymoquinone and aerobic exercise mitigate HFpEF-induced cardiac injury via Apaf1/Cycs axis regulation

**DOI:** 10.3389/fphar.2025.1672570

**Published:** 2025-10-09

**Authors:** Zuowei Pei, Shan Gao, Jin Yang, Chang Liu, Yanyan Pan

**Affiliations:** ^1^ Department of Cardiology, Central Hospital of Dalian University of Technology, Dalian, China; ^2^ Department of Central Laboratory, Central Hospital of Dalian University of Technology, Dalian, China; ^3^ Faculty of Medicine, Dalian University of Technology, Dalian, China

**Keywords:** HFPEF, thymoquinone, aerobic exercise, cardiac damage, Apaf1/Cycs

## Abstract

**Introduction:**

Heart failure with preserved ejection fraction (HFpEF) is a prevalent clinical syndrome associated with high morbidity and mortality. Oxidative stress, apoptosis, and fibrosis are central to its pathophysiology. This study aimed to investigate the synergistic cardioprotective effects of thymoquinone (TQ) and aerobic exercise, with a particular focus on the Apaf1/Cycs-mediated apoptotic pathway.

**Methods:**

Male C57BL/6J mice were randomly assigned to five groups: Control, HFpEF, HFpEF + TQ, HFpEF + exercise, and HFpEF + TQ + exercise. HFpEF was induced by a high-fat diet combined with Nω-Nitro-L-arginine methyl ester (L-NAME) administration for 10 weeks. TQ (50 mg/kg/day) and aerobic exercise (60% of maximal velocity, 5 days/week for 12 weeks) were applied as interventions. *In vitro* HFpEF models were established in H9c2 cardiomyoblast cells treated with L-NAME. Histological and molecular changes were assessed by immunofluorescence, co-immunoprecipitation, and TUNEL assays.

**Results:**

The combined treatment of TQ and aerobic exercise significantly reduced myocardial fibrosis, apoptosis, and oxidative stress compared with either intervention alone. Mechanistically, the combination downregulated the interaction between Apaf1 and Cycs, thereby suppressing cardiomyocyte apoptosis.

**Discussion:**

These findings demonstrate that TQ and aerobic exercise exert synergistic cardioprotective effects in HFpEF by attenuating oxidative stress, fibrosis, and apoptosis through inhibition of the Apaf1/Cycs apoptotic pathway. The results provide new insights into adjunctive therapeutic strategies for HFpEF management.

## Introduction

Heart failure (HF) is a major global public health concern, affecting millions of adults worldwide (Borlaug, 2020). Among its various forms, heart failure with preserved ejection fraction (HFpEF) accounts for approximately 50% of all HF cases and is the most prevalent subtype, associated with considerable morbidity and mortality (Steinberg et al., 2012; Dunlay et al., 2017). Notably, the incidence and prevalence of HFpEF have escalated by approximately 10% per decade, surpassing the rates observed in heart failure with reduced ejection fraction (HFrEF) (Borlaug et al., 2023). Key risk factors for the development of HFpEF include obesity, metabolic dysfunction, and physical inactivity, underscoring the multifactorial nature of its pathogenesis (Pfeffer et al., 2019). Elevated levels of apoptotic markers have been detected in the myocardium of HFpEF patients, suggesting that apoptosis plays a critical role in the loss of functional cardiomyocytes and the development of diastolic dysfunction (Omote et al., 2022).

Thymoquinone (TQ), a bioactive phytochemical extracted from the traditional medicinal plant Nigella sativa, has garnered attention for its potential therapeutic benefits in HFpEF. Previous research by our group has demonstrated that TQ possesses robust antioxidant, anti-inflammatory, and anti-apoptotic properties [6]. TQ may mitigate key pathological features of HFpEF, including oxidative stress, myocardial fibrosis, and endothelial dysfunction. By targeting these mechanisms, TQ emerges as a promising adjunctive therapy aimed at enhancing cardiac function and addressing the complex, multifactorial pathophysiology of HFpEF-a condition for which effective treatment options remain limited in current clinical practice (El Mezayen et al., 2006; Kundu et al., 2014; Jayasooriya et al., 2014). Furthermore, TQ has been shown to reduce oxidative stress, restore redox balance, and preserve cardiomyocyte integrity (Ojha et al., 2015). Mechanistically, TQ preserves mitochondrial integrity by attenuating oxidative stress, thereby inhibiting the activation of the intrinsic apoptotic pathway. Additionally, its anti-inflammatory effects downregulate pro-inflammatory cytokines like TNF-α and IL-6, which are known to induce apoptosis via extrinsic pathways (Abudureyimu et al., 2024).

Aerobic exercise is increasingly recognized as an effective non-pharmacological intervention for managing HFpEF. Regular aerobic training enhances cardiovascular function, reduces systemic inflammation, and improves endothelial health [11]. Clinical studies have shown that aerobic exercise training can increase exercise tolerance and quality of life in HFpEF patients by promoting mitochondrial function, enhancing skeletal muscle efficiency, and decreasing ventricular stiffness. These effects directly address the key pathophysiological mechanisms underlying HFpEF [12]. In the context of rising obesity rates, metabolic disorders, and sedentary lifestyles, regular moderate-intensity continuous training (MICT) offers substantial benefits for cardiovascular health and reduces overall disease mortality. Exercise confers a broad spectrum of cardiovascular advantages, including improved hemodynamics, decreased prevalence of coronary artery disease and cardiomyopathy, and enhanced cardiac reserve capacity and autoregulation (Wu et al., 2019). The Apaf1/Cycs axis plays a central role in the mitochondrial (intrinsic) apoptotic pathway. Upon receiving pro-apoptotic signals, cytochrome c (Cycs), released from mitochondria into the cytoplasm, binds to apoptotic protease activating factor 1 (Apaf1), leading to the formation of the apoptosome. This multi-protein complex recruits and activates procaspase-9, thereby initiating a cascade of downstream caspase activation that culminates in programmed cell death (Judson et al., 2000; Orzáez et al., 2014). Through this pathway, the Apaf1–Cycs interaction serves as a molecular switch for apoptosis, and its dysregulation has been implicated in the pathogenesis of cardiovascular diseases, including heart failure (Štika et al., 2024).

Although both TQ and aerobic exercise have independently demonstrated beneficial effects in reducing oxidative stress and improving cardiac function, respectively, there is a lack of studies investigating the combined therapeutic potential of these interventions in HFpEF. We hypothesize that the synergistic effects of TQ and aerobic exercise could more effectively target the underlying pathophysiological mechanisms of HFpEF, thereby offering enhanced improvements in patient outcomes compared to each intervention alone. This study aims to evaluate the therapeutic efficacy and underlying mechanisms of combined TQ and aerobic exercise treatment in a mouse model of HFpEF, providing insights into a novel multimodal approach for managing this complex and increasingly prevalent condition.

## Materials and methods

### Establishment and treatment of mouse model

Eight-week-old male C57BL/6J mice were purchased from Liaoning Changsheng Biotechnology Co., Ltd. (Liaoning, China). All animals were housed under standard laboratory conditions, including a temperature of 24 °C–26 °C, relative humidity of 40%–60%, and a 12-h light/dark cycle. Following acclimatization, a total of 60 mice were randomly assigned to five experimental groups: Control group (n = 12); HFpEF group (n = 12); HFpEF + TQ group (n = 12); HFpEF + E group (n = 12); HFpEF + TQ + E group (n = 12). Distinct therapeutic interventions were administered to each group according to the study protocol. In HFpEF group, mice were fed a high-fat diet (D12492, provided by Beijing Xiaoshu Youtai Biotechnology Co., LTD.) with 60% energy from fat and provided drinking water containing Nω-Nitro-L-arginine methyl ester hydrochloride (L-NAME; MedChemExpress, New Jersey, United States) at a concentration of 0.5 g/L for 10 weeks. After successful induction of HFpEF, mice in the TQ-treated groups received TQ dissolved in corn oil at a dose of 50 mg/kg daily, and mice in the exercise groups underwent aerobic treadmill training at 60% of maximal running speed (Vmax) for a total of 12 weeks. The body weight of the experiment mice was measured every week. At the end of the experimental period, blood samples were collected from the abdominal aorta and transferred into serum separation tubes. All samples were immediately stored at −80 °C until further analysis. The thigh bone was stripped of muscle. Heart tissue was fixed in 10% formalin and paraffin-embedded for histological observation. The remaining heart tissue was rapidly frozen in liquid nitrogen for histology, qPCR, and Western blot analysis. All animal experiments were performed in accordance with the Guide for the Care and Use of Laboratory Animals and approved by the Ethics Committee of Dalian Municipal Central Hospital.

### Exercise training regimen

The maximum running speed of HFpEF mice in exercise group was measured by treadmill test on XR-PT-10B treadmill (Shanghai Xinsoft Information Technology Co., LTD., Shanghai, China). The test started at 10 m/min with a 0° tilt for 20 min and increased at a rate of 4 m/min/min until exhaustion. Mice were considered exhausted if they remained stationary on the grid for 3 s. The maximum speed achieved during exercise was defined as peak running speed. Before formal training, HFpEF mice underwent a standardized 5-min warm-up at 40% of their respective maximum running speed. The HFpEF + E and HFpEF + TQ + E groups performed continuous endurance training at 60% of their maximum running speed for 21.5 min without rest. After training, a 5-min recovery period on the ground at 40% of maximum running speed was performed, repeated five times per week for 12 weeks.

### Mouse echocardiography

Mice were anesthetized via intraperitoneal injection of 2% tribromoethanol at a dose of 0.01 mL/g. Anesthesia depth was carefully monitored, and chest hair was removed following adequate sedation. The mice were fixed in a supine position on a thermostatic operating table and connected to the electrocardiograph (ECG), and the coupling agent was evenly applied to the forechest and limbs of the mice. A 30 MHz high-frequency ultrasound probe was positioned on the left hemithorax at an approximate angle of 30° to the long axis of the body to obtain a parasternal long-axis view of the left ventricle, ideally visualizing both the mitral and aortic valves. For the short-axis view, the probe was rotated 90° clockwise. B-mode and M-mode echocardiographic images were recorded separately. The early (E) and atrial (A) peak velocities, as well as ejection velocity, were measured using VisualSonics software (Version 1.3.8). Ejection fraction (EF) and fractional shortening (FS) were calculated from M-mode images, and all parameters were averaged over three consecutive cardiac cycles to ensure accuracy.

### Elisa measurements

Blood samples were collected from the abdominal aorta of mice and centrifuged at 3,000 rpm for 10 min to separate the serum. The supernatant was carefully collected and used for subsequent biochemical analyses. Serum levels of N-terminal pro–brain natriuretic peptide (NT-proBNP) were determined using an enzyme-linked immunosorbent assay (ELISA) kit provided by Jianglai Biotechnology Co., Ltd. (Shanghai, China). In addition, the concentrations of superoxide dismutase (SOD) and malondialdehyde (MDA) were measured using colorimetric assay kits obtained from the Nanjing Jiancheng Institute of Biological Engineering (Nanjing, China), in accordance with the manufacturers’ instructions.

### Histological examination of tissue sections

Heart tissue samples were fixed in 4% paraformaldehyde at 4 °C overnight. The following day, tissues were dehydrated through a graded ethanol series (10 min each in 75%, 80%, 90%, 95%, and 100% ethanol), cleared in xylene (two changes, 10 min each), and subsequently embedded in paraffin. Serial sections of 5 μm thickness were prepared from the paraffin-embedded tissues. These sections were deparaffinized by soaking in xylene (two changes, 12 min each), rehydrated through a descending ethanol series (5 min each in 100%, 95%, 85%, and 75% ethanol), and washed in phosphate-buffered saline (PBS) three times for 5 min each. Sections were then subjected to various staining protocols: hematoxylin-eosin (H&E), Masson’s trichrome staining, fluorescein isothiocyanate (FITC)-conjugated wheat germ agglutinin (WGA) staining, Picro Sirius Red staining (PSR), Periodic Acid-Schiff (PAS) staining, and terminal deoxynucleotidyl transferase dUTP nick end labeling (TUNEL) assay. After staining, sections were dehydrated through an ascending ethanol series (5 min each in 75%, 85%, 95%, and 100% ethanol), cleared in xylene (two changes, 12 min each), and mounted with neutral resin. The stained sections were air-dried and histologically analyzed under a BX40 upright light microscope (Olympus, Tokyo, Japan). ImageJ software (NIH, United States) was used to record staining intensity and positivity.

### Transmission electron microscopy

Samples were fixed in 2.5% glutaraldehyde at 4 °C for 48 h, followed by post-fixation in 1% osmium tetroxide for 2 h at room temperature. After washing three times with phosphate-buffered saline (PBS), tissues were dehydrated through a graded ethanol series, embedded in resin, and sectioned into ultrathin slices. Sections were subsequently stained with uranyl acetate and lead citrate and examined using a JEM-1400 transmission electron microscope (JEOL Ltd., Tokyo, Japan).

### Immunohistochemical analysis

The embedding and deparaffinization procedures were performed as described for histological staining. A citric acid/sodium citrate buffer (pH = 6) was prepared and heated in a microwave oven at high power for 3–5 min. The tissue sections were then incubated in the buffer at low heat for 1–2 min, followed by cooling at room temperature for 30 min. After cooling, the sections were washed three times with PBS for 5 min each. Peroxidase activity was blocked by applying a peroxidase-blocking reagent to the tissue for 10 min, followed by three washes with PBS for 5 min each. The sections were then blocked with sheep serum at room temperature for 1 h. Next, they were incubated overnight at 4 °C with the following primary antibodies: rabbit anti-collagen I (1:300, Proteintech, Wuhan, China), rabbit anti-collagen III (1:300, Abcam, Cambridge, United Kingdom), rabbit anti-TGF-β (1:300, Proteintech, Wuhan, China), rabbit anti-MMP7 (1:300, Proteintech, Wuhan, China), rabbit anti-Apaf1 (1:100, Affinity, Jiangsu, China), and rabbit anti-Cycs (1:100, Affinity, Jiangsu, China). The next day, the primary antibodies were removed, and the sections were washed with PBS three times for 5 min each. The tissue was then incubated with the secondary antibody (HRP-conjugated goat anti-rabbit IgG) from the N-Histofine Simple Staining Kit for 1 h at room temperature. Following this, the sections were incubated in 3,3′-diaminobenzidine (DAB, Solarbio Science & Technology, Beijing, China, DA1015) for 10 min to visualize the antigen-antibody complex, and the reaction was stopped by rinsing in distilled water. Hematoxylin staining was performed for 1–2 min, followed by rinsing with running water for 1 h. To differentiate, sections were briefly treated with 1% hydrochloric acid alcohol for 3 s and then rinsed under running water for 3 min. Finally, the slides were dehydrated in a graded ethanol series (5 min each in 75%, 85%, 95%, and 100% ethanol), immersed in xylene (two changes, 12 min each), and mounted with neutral resin. The sections were air-dried at room temperature and examined morphologically using a BX40 upright light microscope (Olympus, Tokyo, Japan). quantification was performed using ImageJ software (NIH, United States).

### Proteomic profiling

Protein extraction was carried out using SDT lysis buffer composed of 4% SDS, 100 mM Tris-HCl, and 1 mM DTT (pH 7.6). Following lysis, protein samples were subjected to Coomassie Brilliant Blue R-250 staining to verify the integrity and distribution of protein bands. For proteomic analysis, samples were analyzed using liquid chromatography–tandem mass spectrometry (LC-MS/MS) on a Q Exactive mass spectrometer (Thermo Fisher Scientific) coupled with an Easy-nLC system. The instrument operated in positive ion mode, utilizing a data-dependent acquisition strategy (Top10 method). Full MS scans were recorded at a resolution of 70,000, while higher-energy collisional dissociation (HCD) MS/MS spectra were acquired at a resolution of 17,500. The normalized collision energy was set to 30 eV. Identification and quantitation of proteins use MaxQuant 1.5.3.17 software to merge and search the original MS data of each sample for identification and quantitative analysis.

### Western blot and immunoprecipitation

Frozen murine heart tissues stored at −80 °C were retrieved and immediately placed into 1.5 mL microcentrifuge tubes preloaded with ice-cold protease and phosphatase inhibitor cocktail. Tissues were finely minced on ice and subsequently lysed via ultrasonic homogenization overnight to ensure thorough protein extraction. The following day, lysates were centrifuged at 12,000 rpm for 40 min at 4 °C. The resulting supernatant was transferred to fresh 1.5 mL tubes, and the total volume was recorded. Protein concentrations were determined using a bicinchoninic acid (BCA) protein assay kit (Beyotime Biotechnology, Shanghai, China). A standard curve was generated using serial dilutions of known protein concentrations (2.000, 1.000, 0.500, 0.250, 0.125, and 0.0625 mg/mL). Absorbance at 562 nm was measured using a microplate reader. Quantified protein samples were aliquoted into new tubes, denatured by boiling at 100 °C for 5 min, and stored at −80 °C until further use. Equal amounts of protein were separated by 10% SDS-PAGE using the Easy PAGE® Color Rapid Gel Preparation Kit (SEVEN, Beijing, China), followed by electrotransfer onto polyvinylidene fluoride (PVDF) membranes (Immobilon, Millipore, Billerica, MA, United States). Membranes were blocked at room temperature for 1 h with 5% non-fat milk prepared in TBST buffer (Tris-buffered saline containing 0.1% Tween-20), and subsequently incubated overnight at 4 °C with the following primary antibodies: Rabbit anti-NPPA (1:2000, Proteintech, Wuhan, China), Rabbit anti-NPPB (1:2000, Proteintech, Wuhan, China), Rabbit anti-BNP (1:500, Abcam, Cambridge, United Kingdom), Rabbit anti-collagen I (1:1000, Proteintech, Wuhan, China), Rabbit anti-collagen III (1:1000, Abcam, Cambridge, United Kingdom),Rabbit anti-TGF-β (1:1000, Proteintech, Wuhan, China), Rabbit anti-MMP7 (1:1000, Proteintech, Wuhan, China), Rabbit anti-Apaf1 (1:1000, Affinity Biosciences, Jiangsu, China), Rabbit anti-Cycs (1:1000, Proteintech, Wuhan, China), Rabbit anti-MT-ATP6 (1:2000, Affinity Biosciences, Jiangsu, China), Rabbit anti-phospho-NF-κB p65 (1:1000, Proteintech, Wuhan, China), Rabbit anti-NF-κB p65 (1:1000, Proteintech, Wuhan, China), Rabbit anti-Caspase-9 (1:800, ABclonal, Wuhan, China), Rabbit anti-Cleaved-caspase-3 (1:1000, Proteintech, Wuhan, China), Rabbit anti -caspase-3 (1:1000, Proteintech, Wuhan, China), Rabbit anti-Bax (1:1000, Proteintech, Wuhan, China) and Rabbit anti-Bcl-2 (1:1000, Proteintech, Wuhan, China).

On the second day, membranes were washed three times with TBST buffer (5 min each wash) to remove unbound primary antibodies. Subsequently, membranes were incubated for 1 h at room temperature with the appropriate horseradish peroxidase (HRP)-conjugated secondary antibodies: anti-rabbit IgG (1:5000) or anti-mouse IgG (1:10000) (Proteintech, Wuhan, China). After secondary antibody incubation, membranes were washed again with TBST and immunoreactive bands were visualized using an enhanced chemiluminescence (ECL) detection system.

Densitometric analysis of protein bands was performed using NIH ImageJ software. Mouse anti-β-actin (1:10000, Proteintech, Wuhan, China) or rabbit anti-GAPDH (1:5000, Proteintech, Wuhan, China) served as internal loading controls. Target protein expression levels were normalized to those of β-actin or GAPDH to ensure equal protein loading across samples.

For immunoprecipitation of anti- Apaf1 and anti Cycs antibodies, 100 μg cell lysate were incubated with 2 μg Cycs and Apaf1 antibody and thereafter, were incubated with Protein A/G-Sepharose beads (Thermo Fisher). Proteins were separated by 10% SDS/PAGE and electrotransferred into nitrocellulose filters. Membranes were incubated with Cycs and Apaf1 antibodies overnight at 4 °C. Membranes were incubated with HRP conjugated anti-rabbit secondary antibody for 1 h at room temperature, and protein bands were visualized with enhanced chemiluminescence reagents (SEVEN, Beijing, China).

### Cell culture

Rat cardiac myoblast H9c2 cells were purchased from Pricella Biotechnology Co., Ltd. (Wuhan, China). Cells were used in the 4-8^th^ passage. Cells were cultured in 10% fetal bovine serum (FBS)-DMEM in a 37 °C, 5% CO_2_ humidified incubator. Cell viability determined by Trypan Blue exclusion was consistently greater than 98%.

### Immunofluorescence

Dewaxing, antigen retrieval, and blocking procedures were performed following standard immunohistochemical protocols. Tissue sections were incubated overnight at 4 °C with a mixture of primary antibodies: rabbit anti-Apaf1 (1:100, Affinity Biosciences, Jiangsu, China) and mouse anti-Cycs (1:100, Proteintech, Wuhan, China), diluted in antibody diluent buffer and applied simultaneously.

The following day, slides were washed three times with phosphate-buffered saline (PBS), 5 min per wash. In the dark, sections were then incubated with species-specific fluorescent secondary antibodies for 1.5 h at room temperature: Alexa Fluor® 488-conjugated anti-rabbit IgG (H + L), F(ab’)2 fragment (1:800, Cell Signaling Technology, MA, United States), and Alexa Fluor® 594-conjugated anti-mouse IgG (H + L), F(ab’)2 fragment (1:800, Cell Signaling Technology). After incubation, sections were rinsed three times in PBS. For nuclear counterstaining, DAPI (Servicebio, Wuhan, China) was applied for 10 min in the dark, followed by three additional PBS washes. The slides were then mounted using an anti-fade fluorescence mounting medium (Servicebio, Wuhan, China).

Images were acquired using a Leica TCS SP8 confocal laser scanning microscope (Leica Microsystems, Wetzlar, Germany), equipped with laser excitation at 405, 488, and 561 nm. All fluorescence images were captured under identical exposure conditions and processed with Leica LAS X software for visualization and analysis.

### Stable overexpression of target genes in cultured cells (OE)

H9c2 cells were transfected with plasmid constructs encoding rat Apaf1 or Cycs, as well as an empty vector (NC-P) serving as a negative control. The final concentration of plasmid DNA used for transfection was 2.5 mg/L. Transfection was carried out using Neofect DNA transfection reagent (Neofect, Beijing, China) according to the manufacturer’s instructions. After 48 h of incubation, cells were harvested for subsequent protein extraction. The Apaf1 and Cycs overexpression plasmids were generated by subcloning the respective rat cDNA sequences into the pUC57 vector (Cax9X Biotech).

### Quantitative real-time PCR

Total RNA was extracted from tissues or the cardiac myoblast using TRIzol reagent (Vazyme; Nanjing China). mRNA (1000 ng) was reversely transcribed into cDNA using MonScript™ RTIII All-in-One Mix (Monad; Suzhou China). The primer sequences and the related SYBR Green probes are shown in Table 1.

**TABLE 1:** List of primer sequences.

Gene	Primer sequences
*m-Naalad2-n-F* *m-Naalad2-n-R* *m-CALCRL-F* *m-CALCRL-R* *m-Bcl2-F* *m-Bcl2-R* *m-Cycs-F* *m-Cycs-R* *m-Afap1-F* *m-Afap1-R* *m-PICALM-F* *m-PICALM-R* *m- NF-kB(P65)-F* *m- NF-kB(P65)-R*	TGCAGCCATATCCCAAAGGA TCCCCACTGCTTCTTCAACAG GGCCTTAGTGGCCACAAATC AAGTGCTGCTTCTCCGCAAA CGTCGCTACCGTCGTGACTT CCCCACCGAACTCAAAGAAG GGCTGCTGGATTCTCTTACACA GCCCTTTCTCCCTTCTTCTTAATT AAGCTGCTCTGCGTCATCAA GGTGATTTTCAGCTCGTGTTTCT CAGGCAGCATTAGAGGAAGAACA TCAATGGCTGGTGCAGTCAT CCTCCAACCCGGCGTATT GTTTGAGATCTGCCCTGATGGTA
*m-Caspase3-F* *m-Caspase3-R*	GAGGAGATGGCTTGCCAGAA CTTGTGCGCGTACAGCTTCA
*m-Bax-F* *m-Bax-R* *m-GAPDH-F* *m-GAPDH-R* *m-Rnf31-F* *m-Rnf31-R* *m-IRP1-F* *m-IRP1-R* *m-FTH1-F* *m-FTH1-R*	CCAAGAAGCTGAGCGAGTGTCT TGAAGTTGCCATCAGCAAACA GCCACCCAGAAGACTGTGGAT GGAAGGCCATGCCAGTGA CCGCCCTGACCTCACTGA GGGCGCACCACAAGAACT GTGCGACAGCAGCCTTCTTC CTGGGTCTTGAGAGGTGTCGTT GCATGCCGAGAAACTGATGA AGTGACTGATTCACACTCTTTTCCA

### Gene silencing via RNA interference

H9c2 cells were seeded into 6 cm culture dishes and transfected when cell confluency reached approximately 60%. Gene silencing was performed using small interfering RNA (siRNA) targeting Apaf1 or Cycs. Specific siRNA sequences were designed and synthesized, and transfections were carried out using a commercial siRNA transfection reagent (Cax9X Bioengineering Co., Ltd., China) in accordance with the manufacturer’s protocol. The efficiency of gene knockdown was confirmed by Western blot analysis. The siRNA sequences used for targeting Apaf1 and Cycs are listed in Table 2.

**TABLE 2:** List of siRNA target sequences.

Gene	siRNA target sequences
*Apaf1-F-342* *Apaf1-R-342* *Cycs-F-130* *Cycs-R-130* *Rat GAPDH* *Rat GAPDH*	GAAGUUUAUCGACAAGCAATT UUGCUUGUCGAUAAACUUCTT GCUGCUGGAUUCUCUUACATT UGUAAGAGAAUCCAGCAGCTT AGAAUGGGAAGCUUGUCAUCAATT UUGAUGACAAGCUUCCCAUUCUTT

### Co-immunoprecipitation (CO-IP)

H9c2 cells were treated with different treatments, the cells were collected, the cell extracts were prepared, the antibodies were fixed on the surface of protein A/G AGAR magnetic beads (Thermo Fisher Scientific, Massachusetts, United States), the target protein was captured, and then co-immunoprecipitation was performed.

### MTS colorimetric method for cell viability

Logarithmic-phase H9c2 cells were harvested, and the cell concentration of the suspension was adjusted. After counting, 100 μL of the cell suspension (containing 10,000 cells) was added to each well of a 96-well flat-bottom plate, creating a monolayer at the bottom of the wells. The plate was then incubated in a 5% CO_2_ incubator at 37 °C. On the second day, after serum-starvation for 4 h, the culture medium was aspirated, and cells were treated with a series of L-NAME (MedChemExpress, HY-18729A, New Jersey, United States) concentrations. Cells were incubated for an additional 48 h under standard culture conditions. Subsequently, 20 μL of MTS reagent (Promega, G3582, Beijing, China) was added to each well in the dark, followed by a 2-h incubation at 37 °C. Absorbance was measured at 490 nm using a microplate reader. Cell viability was calculated as a percentage of the control group using the corresponding optical density (OD) values. Each condition was tested in triplicate, and results were statistically analyzed.

### Data analysis and statistical evaluation

All values are presented as mean ± standard error of the mean (SEM). Statistical analyses were performed using SPSS software version 23.0 (SPSS Inc., Chicago, IL, United States). Comparisons between groups were conducted using one-way ANOVA, followed by Tukey’s *post hoc* test. A P-value of less than 0.05 was considered statistically significant.

## Results

### Comprehensive metabolic profiling

**FIGURE 1:**
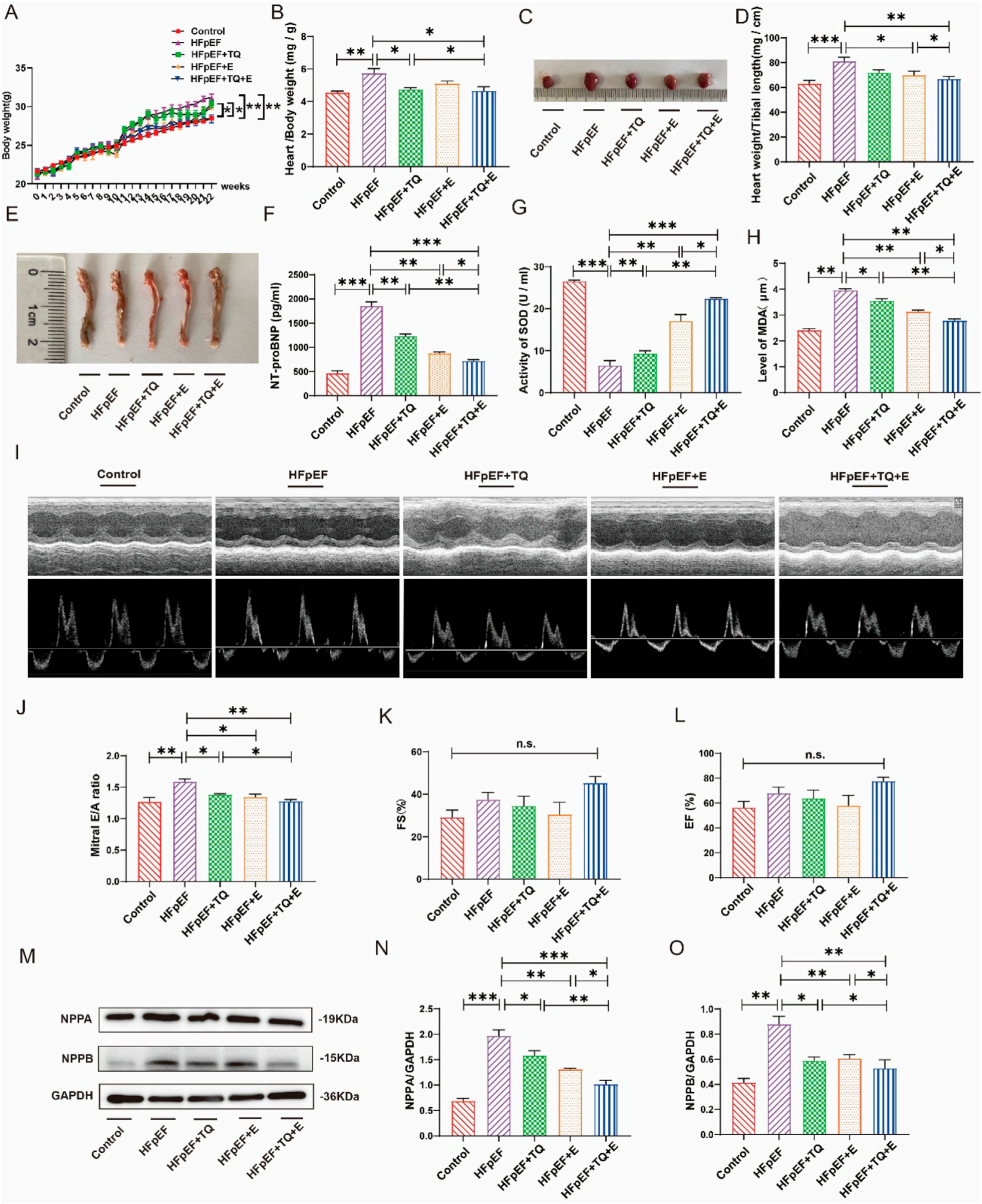
Metabolic data of each group after 12 weeks of different treatment. **(A)** Changes of modeling body weight of mice in each group. **(B)** Quantitative evaluation of the heart weight-to-body weight ratio was performed across all experimental groups. **(C)** Mice heart representatives in each group. **(D)** Quantitative analysis of heart weight/tibial length in each group. **(E)** Tibial length of mice in each group. **(F)** Elisa analysis of NT-proBNP in serum of mice in each group. **(G)** SOD levels in the serum of mice in each group. **(H)** MDA levels in the serum of mice in each group. **(I–L)** The systolic function of mice in each group, representative diagram of EA peak, Ejection Fraction (EF), and Fractional Shortening (FS). **(M–O)** Expression levels of NPPA and NPPB proteins in the heart tissues of mice in each group. (Means ± SEM, Body weight, n = 12; Heart weight/body weight and heart weight/tibial length, n = 6; SOD and MDA levels, n = 5; systolic function and NPPA and NPPB proteins levels, n = 3; *p < 0.05, **p < 0.01, ***p < 0.001, Control group vs. HFpEF group; HFpEF group vs. HFpEF + TQ + E group; HFpEF group vs. HFpEF + E group; HFpEF group vs. HFpEF + TQ group; HFpEF + TQ group vs. HFpEF + TQ + E group; HFpEF + E group vs. HFpEF + TQ + E group; n.s., no significant).

Figure 1 presents the metabolic parameters across the five experimental mouse groups subjected to various treatments. Mice in the HFpEF group exhibited significantly elevated body weight, heart-to-body weight ratio, and heart-to-shin length compared to the control group (*p* < 0.01, *p* < 0.01, *p* < 0.001) (Figures 1A–E). TQ treatment alone (*p* < 0.05, *p* < 0.05) and exercise therapy alone (*p* < 0.05, *p* < 0.05) have a certain therapeutic effect on HFpEF. Notably, the combination therapy of thymoquinone and aerobic exercise (TQ + E) resulted in the most pronounced reductions in these parameters (*p* < 0.01, *p* < 0.05, *p* < 0.01), surpassing the effects observed with TQ (*p* < 0.05, *p* < 0.05) or exercise alone (*p* < 0.05, *p* < 0.05). Serum levels of N-terminal pro-brain natriuretic peptide (NT-proBNP) were assessed via ELISA and were markedly increased in the HFpEF group relative to controls (*p* < 0.001), all three treatments reduced NT-proBNP levels to varying degrees (*p* < 0.01, *p* < 0.01, *p* < 0.001). Treatment with the TQ + E combination significantly decreased NT-proBNP levels more effectively than either TQ (*p* < 0.01) or exercise (*p* < 0.05) individually (Figure 1F). Antioxidant capacity was evaluated by measuring serum superoxide dismutase (SOD) activity. The HFpEF group demonstrated a significant reduction in SOD levels compared to the control group (*p* < 0.001). However, all three treatments increased SOD levels in HFpEF (*p* < 0.01, *p* < 0.01, *p* < 0.001). Administration of TQ + E markedly restored SOD activity, achieving higher levels than those seen with TQ (*p* < 0.01) or exercise (*p* < 0.05) alone (Figure 1G). Lipid peroxidation was assessed by measuring serum malondialdehyde (MDA) concentrations. The HFpEF group showed significantly elevated MDA levels (*p* < 0.01), three treatments reduced MDA levels (*p* < 0.05, *p* < 0.01, *p* < 0.01), whereas the TQ + E treatment substantially lowered MDA levels compared to the HFpEF group and was more effective than either intervention alone (*p* < 0.01, *p* < 0.05) (Figure 1H). Echocardiographic evaluation revealed a significant increase in the E/A ratio in the HFpEF group compared to controls (*p* < 0.01), indicative of diastolic dysfunction. The TQ, E and TQ + E combination therapy significantly improved the E/A ratio (*p* < 0.05, *p* < 0.05, *p* < 0.01), more so than TQ (*p* < 0.05) alone, suggesting enhanced diastolic function (Figures 1I,J). Fractional shortening (FS) and ejection fraction (EF) measurements did not show significant differences among the five groups, indicating preserved systolic function across all treatments (Figures 1K,L).

Western blot analyses of natriuretic peptide A (NPPA) and natriuretic peptide B (NPPB) in heart tissues revealed that HFpEF induction significantly upregulated both NPPA (*p* < 0.001) and NPPB (*p* < 0.01) expression compared to controls. TQ (*p* < 0.5, *p* < 0.5), E (*p* < 0.01, *p* < 0.01) and TQ + E (*p* < 0.001, *p* < 0.01) all improved NPPA and NPPB in the HFpEF group. Treatment with TQ + E substantially attenuated the expression levels of NPPA and NPPB more effectively than either TQ (*p* < 0.01, *p* < 0.05) or exercise (*p* < 0.05, *p* < 0.05) alone (Figures 1M–O), highlighting the synergistic effect of the combined therapy on reducing cardiac stress markers.

### Histopathological alterations in myocardial tissue

**FIGURE 2:**
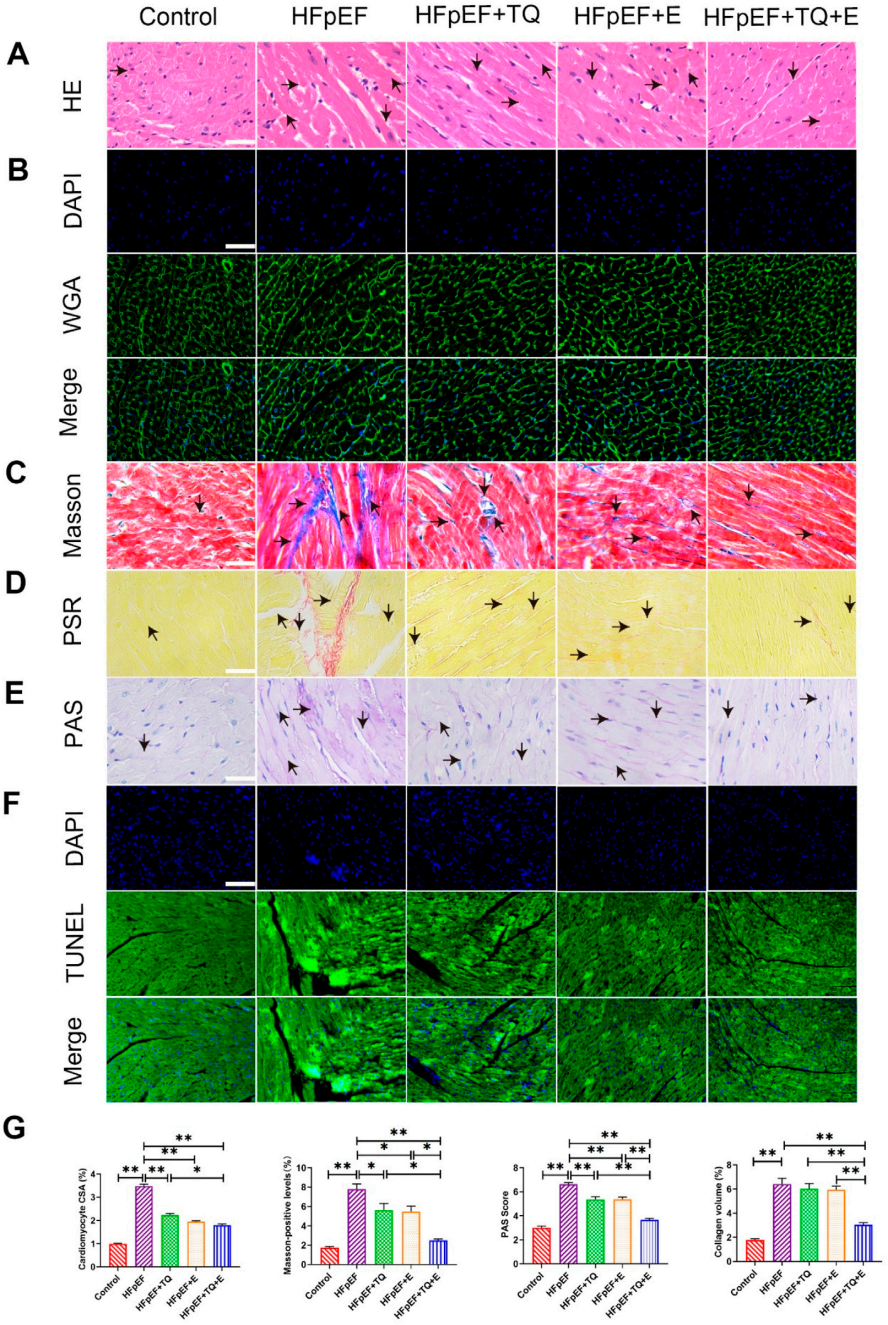
Histopathological changes of cardiac injury in mice treated with HFpEF. **(A)** HE staining of a 40X enlarged section of heart tissue, with arrows indicating positively stained cells. **(B)** Quantitative analysis of cardiomyocyte CSA by WGA staining. **(C)** Masson staining of 40X enlarged sections of heart tissue, arrows indicate the areas where myocardial collagen deposition (fibrosis) occurs. **(D)** Siriusred 40X enlarged section of heart tissue with arrows indicating positively stained cells. **(E)** ×40 magnification of cardiac tissue section PAS, arrows indicating positive stained cells. **(F)** TUNEL staining of a 40X enlarged section of heart tissue, with arrows indicating positive areas. **(G)** Cardiomyocyte CSA, myocardial tissue glycogen deposition, and myocardial collagen deposition were quantitatively analyzed by WGA, Masson tricolor, PAS and PSR staining. (Means ± SEM, n = 3; *p < 0.05, **p < 0.01, Control group vs. HFpEF group; HFpEF group vs. HFpEF + TQ + E group; HFpEF group vs. HFpEF + E group; HFpEF group vs. HFpEF + TQ group; HFpEF + TQ group vs. HFpEF + TQ + E group; HFpEF + E group vs. HFpEF + TQ + E group).

HE staining for tissue morphology, Masson’s trichrome staining for collagen, fluorescein isothiocyanate (FITC)-coupled WGA staining for lectin, PAS staining for glycogen, PSR staining for collagen, and TUNEL staining for apoptosis were used to detect myocardial damage, as shown in Figure 2. HE and WGA staining revealed inflammatory cell infiltration and cardiomyocyte hypertrophy. The HFpEF group exhibited myocardial hypertrophy and a significant increase in the cross-sectional area (CSA) of myocardial cells when compared to the control group (*p* < 0.01). TQ (*p* < 0.01), E (*p* < 0.01) and TQ + E (*p* < 0.01) significantly attenuated myocardial injury. Nevertheless, the cardiac damage factor levels in the HFpEF + TQ + E group were significantly lower than those in the HFpEF + TQ (*p* < 0.05) (Figures 2A,B,G). Masson and PSR staining results showed that, compared with the control group, the fibrosis level of cardiac tissue in the HFpEF group was significantly increased (*p* < 0.01, *p* < 0.01), All three treatments have improved the levels of fibrosis in Masson (*p* < 0.05, *p* < 0.05, *p* < 0.01). whereas the fibrosis level in the TQ + E combined treatment group was significantly improved and to a greater extent than in the TQ (*p* < 0.05, *p* < 0.01) and E (*p* < 0.05, *p* < 0.01) individual treatment groups (Figures 2C,D,G). PAS results showed severe glycogen deposition in the HFpEF group (*p* < 0.01). TQ (*p* < 0.01), E (*p* < 0.01) and TQ + E (*p* < 0.01) treatment all improved glycogen deposition in HFpEF group. The improvement was more pronounced with the combination treatment than with TQ (*p* < 0.01) or E alone (*p* < 0.01) (Figures 2E,G). TUNEL staining results showed that, compared with that in the control group, the apoptosis level of cardiac tissue in the HFpEF group was significantly increased, whereas that in the TQ + E combined treatment group was significantly decreased, with an improvement effect better than that in the TQ and E individual treatment groups (Figure 2F). The results showed that the combined TQ + E treatment was more effective.

### Combined TQ and aerobic exercise attenuate myocardial fibrosis in HFpEF mouse models

**FIGURE 3:**
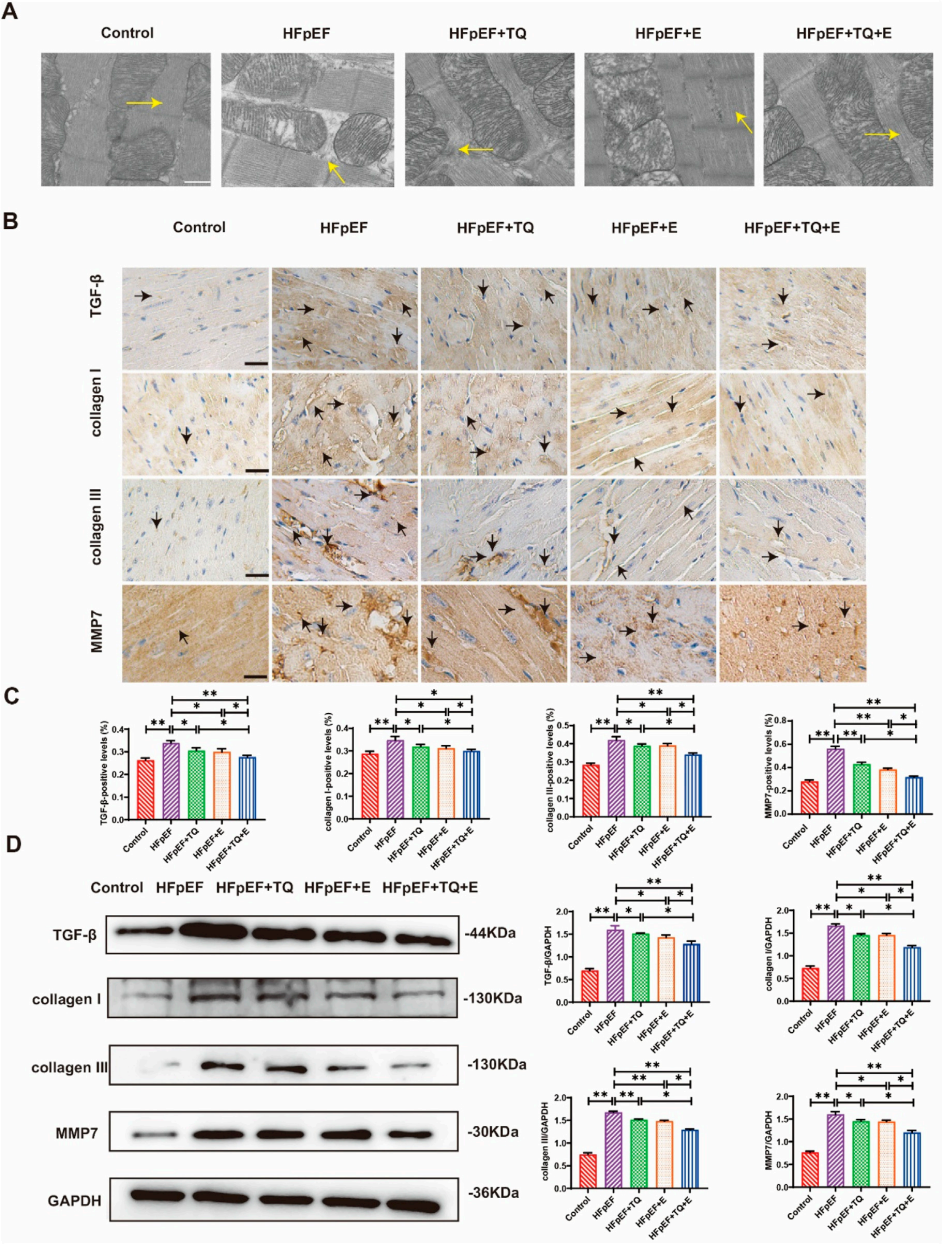
Effects of thymoquinone (TQ) combined with aerobic exercise **(D)** on myocardial fibrosis in HFpEF mice. **(A)** Transmission electron microscopy (TEM) images of sarcomere ultrastructure of HFpEF mice; n = 3. The arrow points to the myotome. Scale = 0.5 µm. **(B)** Immunohistochemical 40X staining results of type I collagen, type Ⅲ collagen, TGF-β and MMP7. The arrows indicate positively stained cells. **(C)** Expression levels of TGF-β, collagen I, collagen Ⅲ, and MMP7 proteins in the heart tissues of mice in each group. (Means ± SEM, n = 3; *p < 0.05, **p < 0.01, Control group vs. HFpEF group; HFpEF group vs. HFpEF + TQ + E group; HFpEF group vs. HFpEF + E group; HFpEF group vs. HFpEF + TQ group; HFpEF + TQ group vs. HFpEF + TQ + E group; HFpEF + E group vs. HFpEF + TQ + E group).

The myotome is the basic unit of myofibrils. Transmission electron microscopy analysis revealed that the myocardial myotome arrangement in HFpEF mice was disorganized or disappeared whereas these changes improved after treatment, among which the improvement observed in the TQ + E group was the most significant (Figure 3A). For further confirming that TQ + E treatment has the most significant effect on myocardial tissue improvement in HFpEF mice, the myocardial tissue was immunohistochemically stained with antibodies against collagen I, collagen Ⅲ, MMP-7, and TGF-β (Figure 3B). The results showed that myocardial collagen deposition and fibrosis levels were significantly higher in the HFpEF group than in the control group (*p* < 0.01, *p* < 0.01, *p* < 0.01, (*p* < 0.01). TQ (*p* < 0.05, *p* < 0.05, *p* < 0.01, *p* < 0.05), E (*p* < 0.05, *p* < 0.05, *p* < 0.01, *p* < 0.05), and TQ + E (*p* < 0.05, *p* < 0.01, *p* < 0.01, *p* < 0.01) improved the protein levels of collagen I, collagen III, MMP-7, and TGF-β, respectively. The improvement of myocardial fibrosis in the HFpEF + TQ + E group was significantly greater than that in the HFpEF + TQ (*p* < 0.05, *p* < 0.05, *p* < 0.05, *p* < 0.05) and HFpEF + E (*p* < 0.05, *p* < 0.05, *p* < 0.05, *p* < 0.05) groups (Figure 3C). Western blot analysis revealed that HFpEF markedly increased the expression of collagen I, collagen Ⅲ, MMP-7, and TGF-β compared with controls (*p* < 0.05, *p* < 0.01, *p* < 0.01, *p* < 0.01). Both TQ (*p* < 0.05, *p* < 0.01, *p* < 0.05, *p* < 0.05) and exercise (*p* < 0.05, *p* < 0.01, *p* < 0.01, *p* < 0.01) significantly attenuated these elevations, while the combined TQ + E treatment produced the greatest improvement (*p* < 0.01, *p* < 0.01, *p* < 0.01, *p* < 0.01), with effects significantly superior to either TQ (*p* < 0.05, *p* < 0.05, *p* < 0.05, *p* < 0.05) or exercise alone (*p* < 0.05, *p* < 0.05, *p* < 0.05, *p* < 0.05) (Figure 3D). Overall, these results demonstrated that TQ combined with aerobic exercise training significantly improved myocardial fibrosis in HFpEF mice.

### Comparative proteomic analysis of cardiac injury across HFpEF treatment groups

**FIGURE 4:**
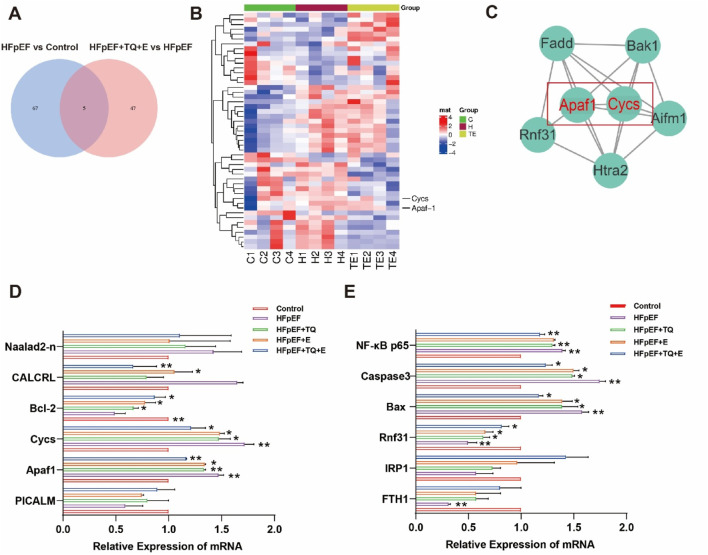
Apoptosis-related factors were significantly enriched in myocardial tissue of HFpEF mice after TQ combined with aerobic exercise. **(A)** Venn diagram. **(B)** Heat map of myocardial tissue. **(C)** The interaction of differential proteins in mouse myocardium was analyzed. **(D,E)** mRNA Relative Expression of myocardial tissue of mice in each group. (Means ± SEM, n = 3; *p < 0.05, **p < 0.01, Control group vs. HFpEF group; HFpEF group vs. HFpEF + TQ + E group; HFpEF group vs. HFpEF + E group; HFpEF group vs. HFpEF + TQ group; HFpEF + TQ group vs. HFpEF + TQ + E group; HFpEF + E group vs. HFpEF + TQ + E group).

Previous studies have shown that TQ combined with moderate-intensity continuous exercise is more effective than monotherapy in HFpEF mice. In order to investigate the factors influenced by cardiac injury in HFpEF mice undergoing exercise training, we performed a proteomic analysis. Differentially expressed proteins were analyzed in mice from the control, HFpEF, and HFpEF + TQ + E groups (Figure 4A). Based on the results of the proteomic analysis, Figure 4B shows a heat map of the genes associated with cardiovascular disease identified by microarray screening. Gene mapping and KEGG pathway analysis of the proteomic data revealed marked alterations in the expression of cardiovascular-related genes across the control, HFpEF, and HFpEF + TQ + E groups (Supplementary Figures S1–S3). The most significant interaction between Apaf1 and Cycs was identified by verifying the interaction levels between different proteins (Figure 4C). The combination of TQ and moderate-intensity continuous exercise has been suggested to play an important role in the course of ejection fraction-preserved heart failure. In addition to examining the expression levels of cardiovascular-related proteins, we also investigated the expression of apoptosis-associated proteins, including phosphatidylinositol-binding clathrin assembly protein (PICALM), calcitonin receptor-like protein (CALCRL), Apaf1, and Naalad2-n in HFpEF mice. The expression of apoptosis-related genes and proteins, including CALCRL, Apaf1, Cycs, Bcl-2 and Rnf31, as well as the downstream markers NF-κB p65, Bax and caspase-3, was significantly altered in HFpEF mice compared with controls (*p* < 0.01, *p* < 0.01, *p* < 0.05, *p* < 0.05, *p* < 0.05, *p* < 0.01, *p* < 0.05, *p* < 0.05). Both TQ (*p* < 0.01, *p* < 0.05, *p* < 0.05, *p* < 0.05, *p* < 0.01, *p* < 0.05, *p* < 0.05) and exercise (*p* < 0.05, *p* < 0.05, *p* < 0.05, *p* < 0.05, *p* < 0.05, *p* < 0.05) intervention partially normalized these changes, while the combined TQ + E treatment produced the most pronounced improvement (*p* < 0.01, *p* < 0.01, *p* < 0.05, *p* < 0.05, *p* < 0.05, *p* < 0.01, *p* < 0.05, *p* < 0.05). Consistent with the proteomic results, the expression of key apoptotic regulators Apaf1 and Cycs followed a similar trend across groups (Figures 4D,E), further supporting that TQ combined with exercise notably attenuated apoptosis in HFpEF mice.

### Combined TQ and aerobic exercise suppress Apaf1/Cycs-mediated cardiomyocyte apoptosis in HFpEF mice

**FIGURE 5:**
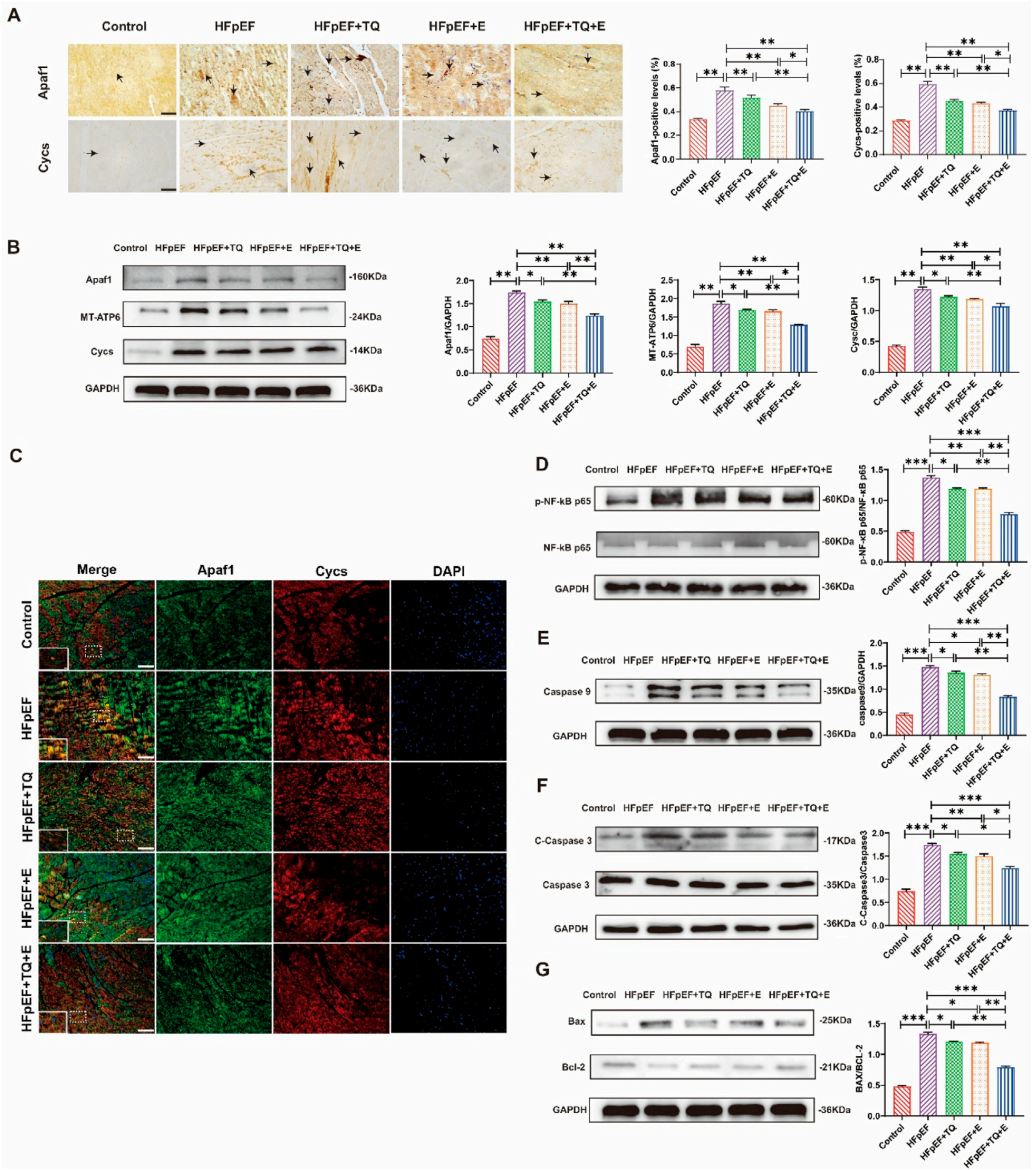
TQ combined with aerobic exercise training significantly improved myocardial cell apoptosis. **(A)** Representative immunohistochemical images depicting Apaf1 and Cycs expression in myocardial tissue sections. Images were captured at ×40 magnification, with arrows indicating regions of positive immunoreactivity. **(B)** Protein expression levels of Apaf1, Cycs, and MT-ATP6 in murine cardiac tissue. **(C)** Double immunofluorescence staining at ×40 magnification was employed to visualize the localization and co-expression of Apaf1 and Cycs in murine myocardial tissue. **(D)** Expression levels of p-NF-κB p65/NF-κB p65 proteins in the heart tissues of mice. **(E)** Expression levels of Caspase9 proteins in the heart tissues of mice. **(F)** Expression levels of Cleaved-Caspaase3/Caspase3 proteins in the heart tissues of mice. **(G)** Expression levels of Bax/Bcl-2 proteins in the heart tissues of mice. (Means ± SEM, n = 3; *p < 0.05, **p < 0.01, ***p < 0.001, Control group vs. HFpEF group; HFpEF group vs. HFpEF + TQ + E group; HFpEF group vs. HFpEF + E group; HFpEF group vs. HFpEF + TQ group; HFpEF + TQ group vs. HFpEF + TQ + E group; HFpEF + E group vs. HFpEF + TQ + E group).

As shown in Figure 5A, the expression of apoptotic factors, Apaf1 and Cycs, screened by proteomics and qPCR in cardiac tissues, was examined by immunohistochemical staining. The expression levels of Apaf1 and Cycs were markedly elevated in the HFpEF group compared with the Control group (both *p* < 0.01). Treatment with TQ (both *p* < 0.01) or exercise (both *p* < 0.01) alone significantly reduced their expression, whereas the combined TQ + E treatment produced the greatest reduction (both p < 0.01), with effects significantly superior to either TQ (both *p* < 0.01) or E (both *p* < 0.05) monotherapy (Figure 5A). Western blot analysis demonstrated that the expression levels of Apaf1 and Cycs were markedly increased in the HFpEF group compared with the Control group (both *p* < 0.01). Treatment with TQ (both *p* < 0.05) or exercise (both *p* < 0.01) alone partially attenuated these elevation, whereas the combined TQ + E treatment resulted in the most pronounced reduction (both *p* < 0.01), with effects significantly greater than those observed in the HFpEF + TQ (both *p* < 0.05) and HFpEF + E (*p* < 0.01, *p* < 0.05) groups. MT-ATP6, an ATPase that interacts with Cycs during apoptosome formation by consuming ATP when activated by Apaf1 [15], was highly expressed in the HFpEF group (*p* < 0.01) but significantly downregulated in the TQ + E group (*p* < 0.01). These results suggest that combined therapy effectively inhibits the Apaf1/Cycs/MT-ATP6 apoptotic signaling axis in HFpEF (Figure 5B), consistent with the finding that Apaf1 and Cycs levels were lower in the HFpEF + TQ + E group than in the HFpEF + TQ and HFpEF + E groups. To prove the interaction between Apaf1 and Cycs, we performed double immunofluorescence staining of the two proteins; The results demonstrated a clear colocalization of Apaf1 and Cycs within myocardial tissue, suggesting their potential interaction in the apoptotic signaling pathway. The co-localized fluorescence expression intensity was the highest in the HFpEF group, whereas that in the TQ + E group was decreased significantly (Figure 5C). The HFpEF group exhibited significantly elevated protein expression levels of p-NF-κB p65/NF-κB p65, Caspase-9, Cleaved-Caspase-3/Caspase-3, and Bax/Bcl-2 compared with the Control group (all *p* < 0.001). Treatment with TQ (all *p* < 0.05) or exercise (*p* < 0.05, (*p* < 0.01, *p* < 0.05, *p* < 0.01) alone partially reduced these pro-apoptotic markers, whereas the combined TQ + E intervention produced the most pronounced reduction (all *p* < 0.001), with effects significantly greater than either TQ (*p* < 0.05, *p* < 0.05, p < 0.01, p < 0.05) or E (*p* < 0.05, *p* < 0.05, *p* < 0.01, *p* < 0.05) monotherapy. (Figures 5D–G). Overall, these results showed that the apoptosis factor Apaf1 interacts with Cycs to activate downstream apoptotic pathways, and that the TQ + E combination therapy significantly inhibits this cell apoptosis.

### Combined TQ and aerobic exercise inhibit L-NAME-induced Apaf1/Cycs interaction in H9c2 cells

**FIGURE 6:**
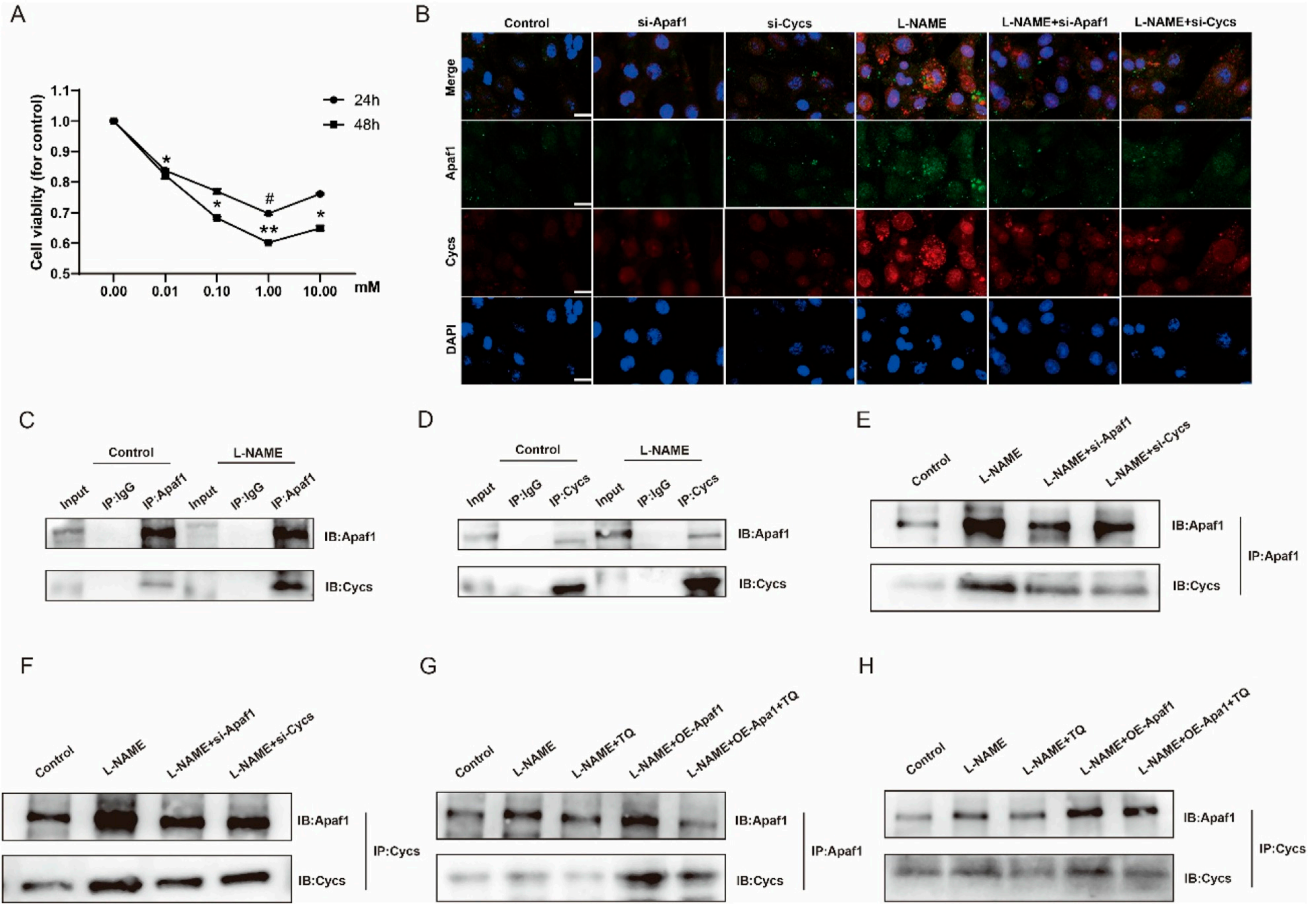
The interaction between Apaf1 and Cycs was enhanced after L-NAME stimulation in the H9c2 cell. **(A)** Effect of L-NAME on H9c2 activity. (Means ± SEM, n = 3; ^#^ *p* < 0.05, 24 h, 0 mM group vs. 1.00 mM group, *p < 0.05, **p < 0.01, 48 h, 0 mM group vs. 0.01 mM group; 0 mM group vs. 0.10 mM group; 0 mM group vs. 1.00 mM group; 0 mM group vs. 10.00 mM group) **(B)** 40X immunofluorescence double staining Apaf1 and Cycs representative image. **(C,D)** Representative images of CO-IP in Apaf1 and Cycs after L-NAME induction. **(E,F)** L-NAME represents the CO-IP after knocking down the gene of Apaf1 or Cycs. **(G,H)** L-NAME induction, overexpression of Apaf1 and TQ processing of CO-IP representative images.

As shown in Figure 6A, the viability of H9c2 cells showed no significant difference among groups after 24 h of L-NAME treatment, except that the 1.00 mM group exhibited a slight but significant reduction compared with the 0 mM group (*p* < 0.05). After 48 h of treatment, cell viability was significantly decreased at 0.01 mM (*p* < 0.05), 0.10 mM (*p* < 0.05), 1.00 mM (*p* < 0.01), and 10.00 mM (*p* < 0.05) compared with the 0 mM group, with the most pronounced inhibitory effect observed at 1.00 mM. Double immunofluorescence staining of Apaf1 and Cycs in H9c2 cells from different treatment groups showed that Apaf1 and Cycs were colocalized in H9c2 cells. Compared with that in the Control group, the expression intensity of Apaf1 and Cycs was stronger, and their co-localization was significantly increased in the L-NAME group. The expression levels of Apaf1 and Cycs were markedly decreased in the L-NAME group subjected to Apaf1 and Cycs silencing compared to the L-NAME group alone, accompanied by a noticeable reduction in their colocalization within the tissue (Figure 6B). CO-IP revealed a significant interaction between Apaf1 and Cycs, and this interaction was more significant in L-NAME-treated H9c2 cells (Figures 6C,D). However, the increased interaction between Apaf1 and Cycs induced by L-NAME was reduced after silencing Apaf1 or Cycs in H9c2 cells (Figures 6E,F). When L-NAME-treated cells overexpressing Apaf1 were treated with TQ, the L-NAME-induced interaction between Apaf1 and Cycs was increased after Apaf1 overexpression, whereas TQ decreased this interaction (Figures 6G,H). In addition, to exclude the off-target effect caused by sirna, we applied shRNA for validation, and the results were the same as those of siRNA (Supplementary Figure S4). These results suggest that Cycs and Apaf1 interact, and that this interaction is enhanced after L-NAME stimulation in H9c2 cells.

### Knockdown of Apaf1 and Cycs suppresses L-NAME-induced apoptosis in H9c2 cells

**FIGURE 7:**
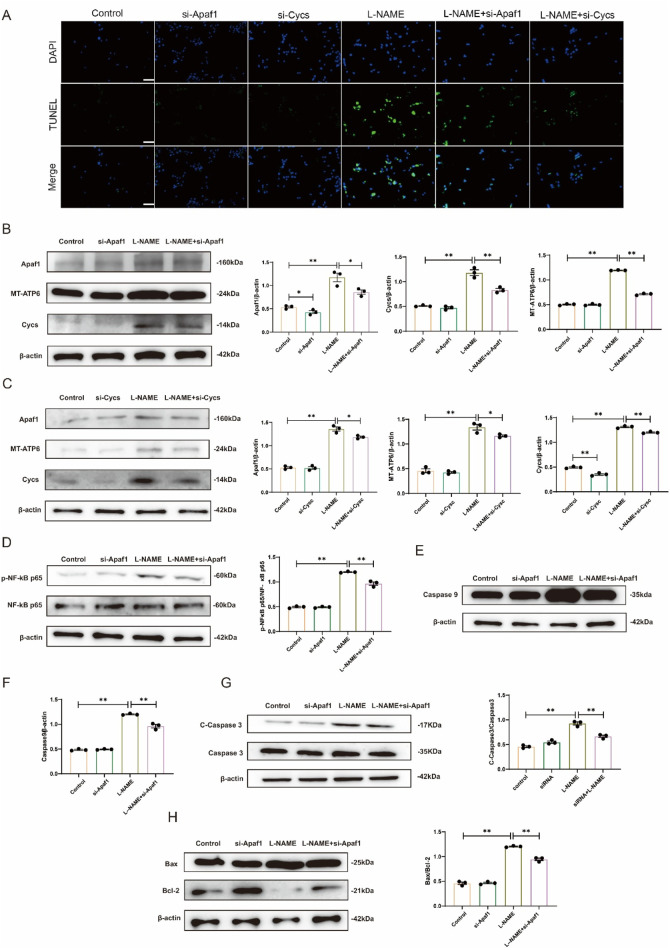
Silencing Apaf1 and Cycs inhibited L-NAME-treated H9c2 apoptosis **(A)** TUNEL staining of a 40X enlarged section of heart tissue, with arrows indicating positive areas. **(B,C)** Representative Western blot images illustrating the protein expression levels of Apaf1, MT-ATP6, and Cycs in H9c2 cells subjected to various treatment conditions. **(D)** Representative Western blot images showing the expression of p-NF-κB p65/NF-κB p65 in Different treatments for H9c2. **(E,F)** Western blot analysis depicting the expression levels of Caspase-9 across various treatment groups in H9c2 cells, accompanied by statistical evaluation of the data. **(G)** Representative Western blot images showing the expression of Cleaved-Caspase3/Caspase3 in Different treatments for H9c2. **(H)** Representative Western blot images showing the expression of Bax/Bcl-2 in Different treatments for H9c2. (Means ± SEM, n = 3; *p < 0.05, **p < 0.01, Control group vs. L-NAME group; L-NAME group vs. L-NAME + si-Apaf1 group; L-NAME group vs. L-NAME + si-Cycs group).

After transfection of Apaf1 siRNA and Cycs siRNA into H9c2 cells, TUNEL staining showed that the apoptosis level in the L-NAME group was significantly lower than that in the untransfected cells treated with L-NAME group. Moreover, the apoptosis level in the L-NAME group with Cycs silencing was significantly lower than that in the untransfected L-NAME group (Figure 7A). Western blot analysis showed that, compared with the Control group, the expression levels of Apaf-1, MT-ATP and Cycs were significantly increased in the L-NAME group (both *p* < 0.01). However, compared with the untransfected L-NAME group, the expression levels of Apaf-1, MT-ATP and Cycs were markedly decreased in the L-NAME + si-Apaf1 group (*p* < 0.05, *p* < 0.01, *p* < 0.01). (Figure 7B). Western blot analysis showed that, compared with the Control group, the expression levels of Apaf-1, MT-ATP and Cycs were significantly increased in the L-NAME group (both *p* < 0.01). However, compared with the untransfected L-NAME group, the expression levels of Apaf-1, MT-ATP and Cycs were markedly decreased in the L-NAME + si-Cycs group (*p* < 0.05, *p* < 0.05, *p* < 0.01) (Figure 7C). Western blot analysis demonstrated that, compared with the untransfected L-NAME group, the protein expression levels of p-NF-κB p65/NF-κB p65 were significantly decreased after Apaf1 silencing (*p* < 0.01). Similarly, the expression levels of Caspase9 (*p* < 0.01), Cleaved-Caspase3/Caspase3 (*p* < 0.01), and Bax/Bcl-2 (*p* < 0.01) were also markedly reduced in the L-NAME + si-Apaf1 group. However, compared with the untransfected L-NAME group, the expression levels of p-NF-κB p65/NF-κB p65, Caspase9 and Cleaved-Caspase3/Caspase3 and Bax/Bcl-2 were markedly decreased in the L-NAME + si-Apaf-1 group (both *p* < 0.01) (Figures 7D–H). The results showed that downregulation of Apaf1 and Cycs significantly improved L-NAME-induced H9c2 apoptosis. Further, downregulation of Apaf1 also inhibited the expression of Cycs and MT-ATP6, while downregulation of Cycs simultaneously inhibited the expression of Apaf1 and MT-ATP6, indicating that Apaf1 and Cycs have a positive reciprocal regulation that requires the participation of MT-ATP6. These results demonstrate that Cycs and Apaf1 interact with each other to activate the downstream apoptotic pathway and induce apoptosis in H9c2 cells after stimulation with L-NAME.

### Overexpression of Apaf1 and Cycs stimulates L-NAME-induced apoptosis in H9c2 cells

**FIGURE 8:**
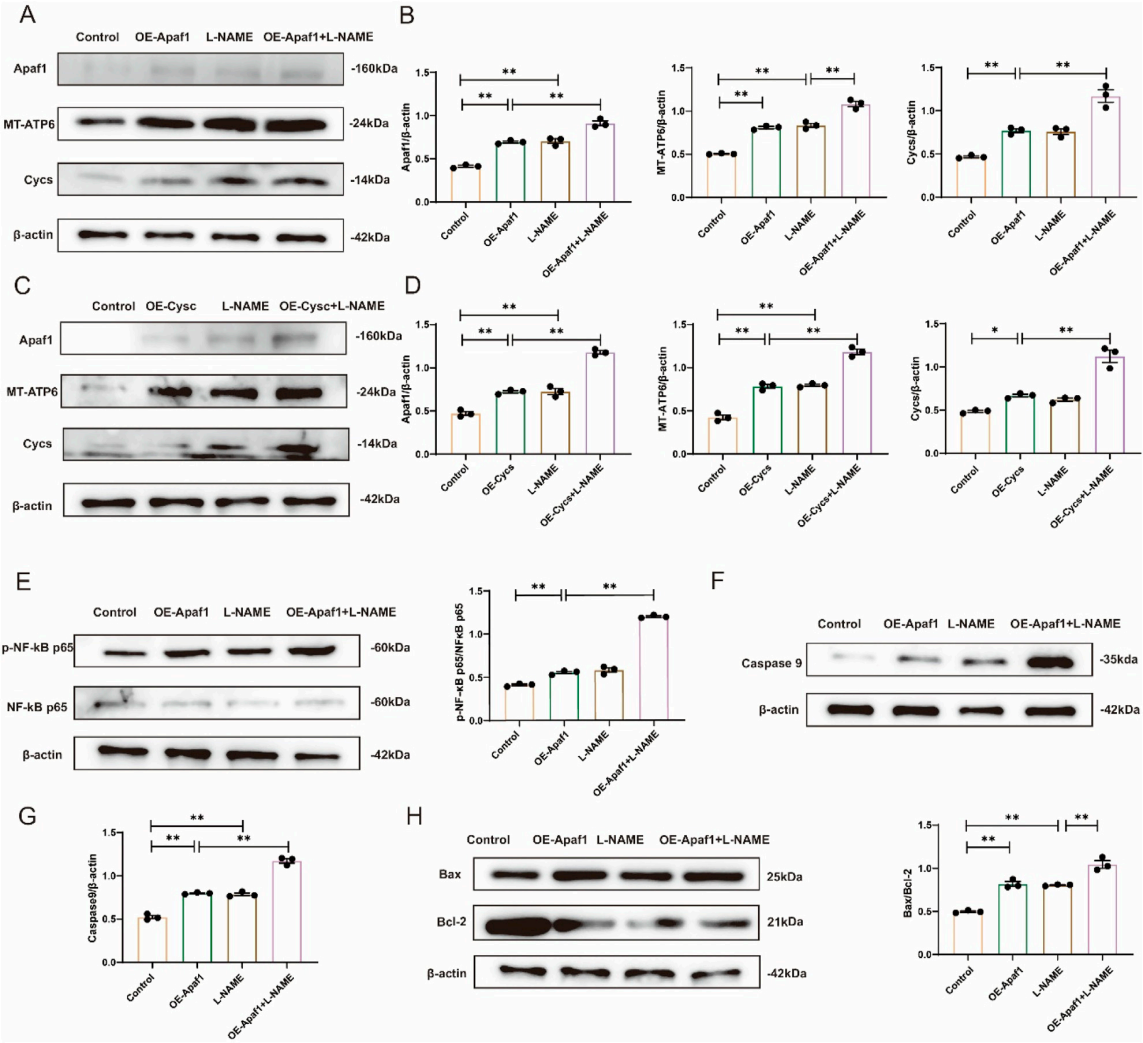
Overexpression of Apaf1 or Cycs stimulates L-NAME-induced apoptosis in H9c2 cells **(A,B)** Representative Western blot images showing the expression of Apaf1, MT-ATP6 and Cycs in different treatments for OE-Apaf1 H9c2. **(C,D)** Representative Western blot images illustrating the expression levels of Apaf1, MT-ATP6, and Cycs in OE-Cycs H9c2 cells subjected to different treatments. **(E)** Representative Western blot images showing the expression of p-NF-κB p65/NF-κB p65 in Different treatments for H9c2. **(F,G)** Representative Western blot images depicting the expression of Caspase-9 in H9c2 cells under various treatment conditions. **(H)** Representative Western blot images showing the expression of Bax/Bcl-2 in different treatments for H9c2. OE, overexpression. (Means ± SEM, n = 3; *p < 0.05, **p < 0.01, Control group vs. L-NAME group; Control group vs. L-NAME + OE-Apaf1 group; Control group vs. L-NAME + OE-Cycs group; OE-Cycs group vs. OE-Cycs + L-NAME group; OE-Apaf1 group vs. OE-Apaf1+L-NAME group; L-NAME group vs. OE-Apaf1+L-NAME group).

Western blot analysis demonstrated that the expression level of Apaf1 was significantly increased in L-NAME-treated H9c2 cells compared with the Control group (*p* < 0.01). Moreover, Apaf1 expression was markedly higher in the L-NAME + OE-Apaf1 group compared with both the OE-Apaf1 group (*p* < 0.01). The expression level of Cycs was significantly elevated in the OE-Apaf1 group compared with the Control group (*p* < 0.01). Furthermore, Cycs expression was markedly increased in the OE-Apaf1+L-NAME group compared with the OE-Apaf1 group (*p* < 0.01). Similarly, the expression of MT-ATP6 was significantly upregulated in the L-NAME group and OE-Apaf1 group compared with the Control group (both *p* < 0.01). Notably, MT-ATP6 expression was further elevated in both the OE-Apaf1+L-NAM groups compared with the OE-Apaf1 group (*p* < 0.01) (Figures 8A,B); when Apaf1 expression increased, Cycs also increased, and *vice versa* (Figures 8C,D). Western blot analysis confirmed the interaction between Apaf1 and Cycs. The expression level of p-NF-κB p65/NF-κB p65, Caspase9 and Bax/Bcl-2 was significantly increased in the L-NAME group compared with the untransfected Control group (both *p* < 0.05). Similarly, the expression of was markedly elevated in the OE-Apaf1+L-NAME group compared with the OE-Apaf1 group (both *p* < 0.01). In addition, the expression of p-NF-κB p65/NF-κB p65, Caspase9 and Bax/Bcl-2 was also significantly increased in the OE-Apaf1 group compared with the Control group (both *p* < 0.01) (Figures 8E–H). These results indicated that the overexpression of Apaf1 or Cycs significantly promoted L-NAME-induced apoptosis in H9c2 cells.

## Discussion

HFpEF remains a major and escalating global health concern, associated with significant morbidity and mortality. In the present study, we provide compelling evidence that the combined intervention of TQ and aerobic exercise training confers robust cardioprotective effects in a murine model of HFpEF. Specifically, this combined therapy significantly alleviates myocardial fibrosis and reduces cardiomyocyte apoptosis. This cardioprotective effect is mediated through the downregulation of the Apaf-1/Cycs axis, a critical pathway involved in apoptosis. *In vitro* experiments using H9c2 cardiomyoblasts revealed that treatment with L-NAME induces apoptosis by upregulating Apaf1, Cycs, and MT-ATP6 expression. The targeted knockdown of Apaf1 and Cycs effectively mitigated L-NAME-induced apoptotic cell death, underscoring the pivotal role of the Apaf-1/Cycs interaction in HFpEF pathogenesis. Furthermore, our findings indicate that the combined therapy of TQ and aerobic exercise not only reduces the expression of these apoptotic mediators but also inhibits their interaction, thereby preventing the activation of downstream apoptotic pathways.

Early intervention with aerobic exercise post-heart failure onset has been shown to improve cardiac function, and our study corroborates these findings (Mi et al., 2021; Böttner et al., 2021; Guo et al., 2022). HFpEF mice subjected to a 12-week regimen of aerobic exercise following L-NAME and high-fat diet induction exhibited significant improvements in cardiac function. These results support the safety and therapeutic efficacy of MICT in alleviating HFpEF-associated cardiac dysfunction, underscoring its value as a non-pharmacological intervention in early-stage HFpEF management.

The pathophysiology of HFpEF is closely associated with chronic myocardial inflammation, characterized by increased monocyte infiltration and elevated levels of pro-inflammatory cytokines (Bachmann et al., 2022).This pro-inflammatory environment contributes to myocardial fibrosis and impaired diastolic function through disruption of collagen homeostasis (Zile et al., 2015). Specifically, cardiac fibroblasts are driven to differentiate into myofibroblasts in response to TGF-β signaling, a process exacerbated by macrophage infiltration (Zhang et al., 2021). Collagen I, collagen III, and TGF-β are key mediators of this fibrotic response. In addition, our study demonstrated that MMP7 expression was markedly increased in HFpEF mice, consistent with its known role in extracellular matrix degradation and pathological remodeling. Both TQ and aerobic exercise reduced MMP7 levels, and the most significant reduction was observed in the combined TQ + E group. These findings suggest that the anti-fibrotic effects of TQ and exercise may be partly mediated through modulation of MMP7, thereby attenuating excessive extracellular matrix turnover and myocardial fibrosis. Our study demonstrates that the combination of TQ and aerobic exercise significantly enhances anti-fibrotic responses compared to either intervention alone, as evidenced by reduced expression of these fibrotic markers.

Persistent collagen deposition and the resultant stiffening of the myocardial extracellular matrix lead to maladaptive signaling, cardiomyocyte hypertrophy, necrosis, and replacement fibrosis (Gurtner et al., 2008). These structural alterations exacerbate cardiac dysfunction and contribute to the progression of HFpEF. Apoptosis plays a fundamental role in cardiovascular biology by removing damaged or dysfunctional cells and maintaining tissue homeostasis (Del et al., 2019). However, accumulating evidence indicates that excessive cardiomyocyte apoptosis contributes to pathological remodeling, fibrosis, and contractile dysfunction in HFpEF (Nair, 2020). In this context, the protective effect of TQ observed in our study should be interpreted as the attenuation of pathological, stress-induced apoptosis, rather than the abolition of physiological apoptotic processes. Proteomic analysis in our study revealed a downregulation of apoptosis-related proteins following combined TQ and aerobic exercise treatment, suggesting that the therapeutic efficacy of this combination is partly mediated through the modulation of apoptotic pathways, particularly the Apaf-1/Cycs axis (Travers et al., 2016). The intrinsic apoptotic pathway, regulated by the Bax/Bcl-2 ratio, plays a crucial role in cardiomyocyte survival (Wang et al., 2024). In HFpEF, an increased Bax/Bcl-2 ratio promotes mitochondrial outer membrane permeabilization, facilitating the release of Cycs into the cytoplasm (Xia et al., 2022). Cycs then interacts with Apaf-1 to form the apoptosome, which activates caspase-9 and subsequently initiates the caspase cascade leading to apoptosis (Wang et al., 2020). Additionally, activated caspase-9 can trigger the PKC/NF-κB signaling pathway, further promoting cell death (Zhao et al., 2014). Our findings indicate that the combination therapy of TQ and aerobic exercise significantly reduces the Bax/Bcl-2 ratio, as well as the levels of caspase-9 and phosphorylated NF-κB p65, thereby inhibiting the apoptotic cascade more effectively than either treatment alone.

Mitochondrial dysfunction is a defining feature of HFpEF, contributing to impaired oxidative phosphorylation and ATP depletion in cardiomyocytes. MT-ATP6, a subunit of ATP synthase, is essential for mitochondrial ATP production and has been shown to exert protective effects against myocardial infarction and hyperlipidemia (Wei et al., 2021). In our study, L-NAME stimulation enhanced the interaction between Apaf1 and Cycs, a process facilitated by MT-ATP6, thereby promoting apoptosis. Co-immunoprecipitation (CO-IP) assays confirmed the increased Apaf1/Cycs interaction in H9c2 cells following L-NAME treatment, which was attenuated by the knockdown of either Apaf1 or Cycs.

Upon activation by Apaf-1, MT-ATP6 binds to Cycs in an ATP-dependent manner and functions as a molecular intermediary regulating the Apaf-1/Cycs axis. In this study, CO-IP was used to determine whether Apaf1 and Cycs showed a significant interaction; this interaction was found to be enhanced in H9c2 cells after L-NAME stimulation. In contrast, the interaction between Apaf1 and Cycs decreased after either of these were silenced.

Although chronic inflammation and apoptosis are widely recognized as hallmarks of HFpEF pathophysiology, the present study offers novel mechanistic insights by identifying the Apaf1/Cycs axis as a key mediator of mitochondria-dependent cardiomyocyte apoptosis in HFpEF. Notably, we demonstrate for the first time that the combination of TQ, a natural antioxidant, and aerobic exercise synergistically attenuates HFpEF-induced cardiac injury by disrupting the interaction between Apaf1 and cytochrome c. This dual-targeted intervention not only suppressed apoptotic signaling more effectively than either treatment alone but also alleviated myocardial fibrosis and oxidative stress.

These findings suggest a potentially promising therapeutic strategy for HFpEF and enhance our understanding of the apoptosis-related molecular mechanisms underlying this complex syndrome. Specifically, our data indicate that the cardioprotective effects of TQ combined with aerobic exercise are mediated through inhibition of the Apaf1/Cycs-mediated apoptotic pathway. This combined therapy mitigates oxidative stress and inflammation, preserves mitochondrial integrity, and enhances cardiomyocyte survival.

However, it is important to note that these findings have yet to be validated in clinical samples, and their translatability to human HFpEF patients remains uncertain. Future studies should aim to identify specific molecular targets downstream of Apaf1 inhibition and evaluate the clinical efficacy of TQ and aerobic exercise as a combinatory therapeutic approach. Such investigations will provide deeper insight into the mechanistic basis of this intervention and its potential application in clinical settings.

In conclusion, this study provides compelling evidence supporting the synergistic cardioprotective effects of TQ and aerobic exercise in the treatment of HFpEF. The therapeutic benefit is largely attributable to modulation of the Apaf1/Cycs signaling axis, a pivotal regulator of cardiomyocyte apoptosis. These findings lay the groundwork for future exploration of novel molecular targets and the development of innovative therapies aimed at improving clinical outcomes in HFpEF.

## Data Availability

The original contributions presented in the study are included in the article/Supplementary Material, further inquiries can be directed to the corresponding author.

## Supplementary material

SUPPLEMENTARY FIGURE S1 Volcanic map of myocardial tissue of mice.

SUPPLEMENTARY FIGURE S2 Bubble map of KEGG pathway in myocardial tissue of mice.

SUPPLEMENTARY FIGURE S3 Bubble map of GO enrichment pathway in myocardial tissue of mice.

## Glossary

HFpEF: Heart failure with preserved ejection fraction
TQ: Thymoquinone
L-NAME: NG-Nitroarginine methyl ester hydrochloride
HF: Heart failure
HFrEF: Ejection fraction-reducing HF
COX2: cyclooxygenase-2
Nrf2: Nuclear factor erythroid 2–related factor 2
PI3K/AKT: Phosphatidylinositol 3-kinase/protein kinase B
NF-Κb: Nuclear factor kappa-light-chain-enhancer of activated B
MICT: Moderate-intensity continued exercise
ECG: Electrocardiograph
EF: Ejection Fraction
FS: Fractional Shortening
HE: Stained with hematoxylin-eosin
Masson: Masson’s trichrome staining
WGA: fluorescein isothiocyanate (FITC)-labeled wheat germ agglutinin
PSR: Picro Sirius Red Stain
PAS: Periodic Acid-Schiff stain
TUNEL: Terminal dexynucleotidyl transferase(TdT)-mediated dUTP nick end labeling
SOD: Superoxide dismutase
Cycs: Cytochrome c
PKC-teta: Protein kinase C-teta
