# Supplementary figures and images for: Thymoquinone and aerobic exercise mitigate HFpEF-induced cardiac injury via Apaf1/Cycs axis regulation
Source: Front Pharmacol. 2025 Oct 9;16:1672570. doi: 10.3389/fphar.2025.1672570 (PMC12546133; doi:10.3389/fphar.2025.1672570)

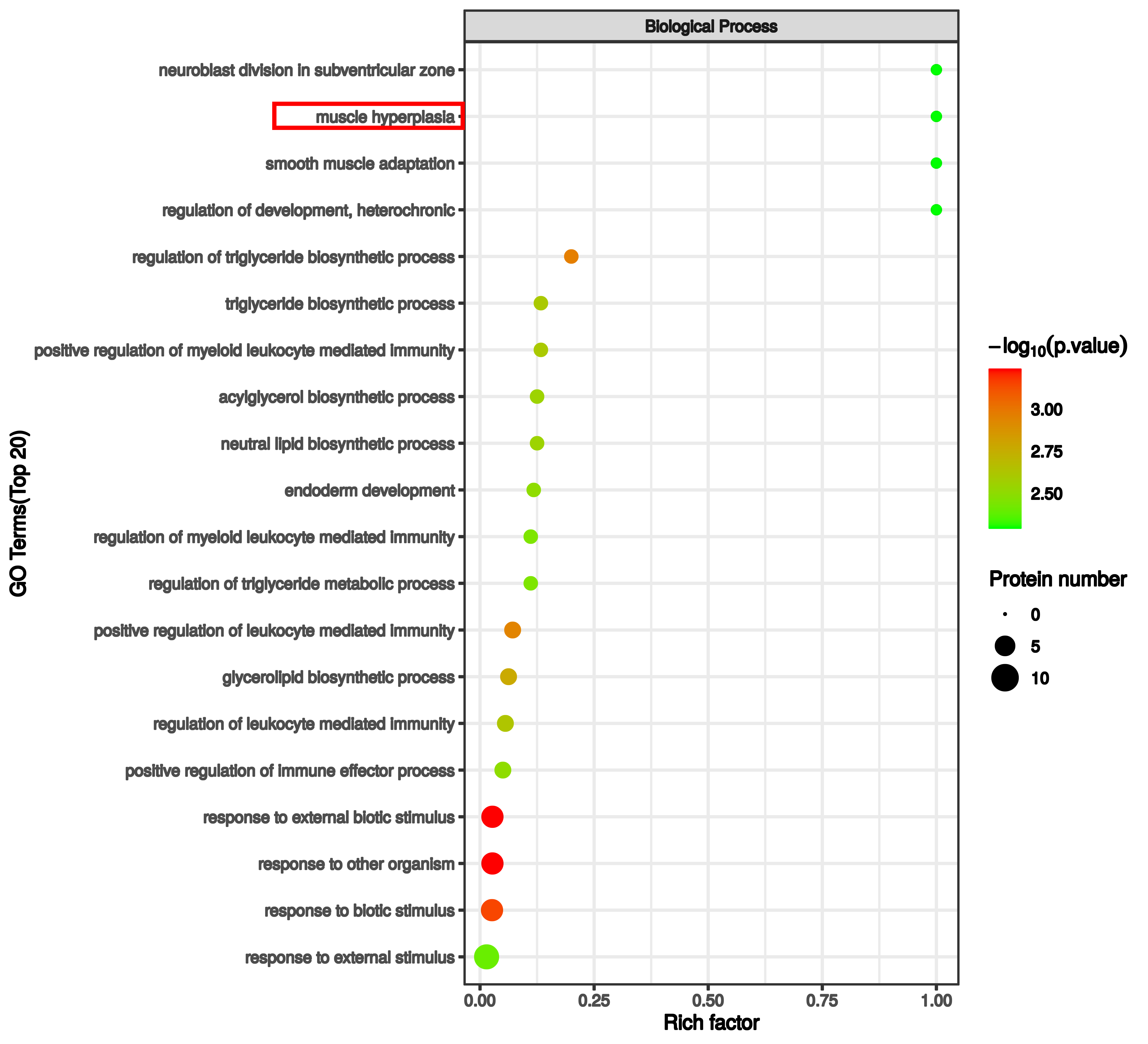

Supplement: Supplementary file 1 [file Image3.jpeg]

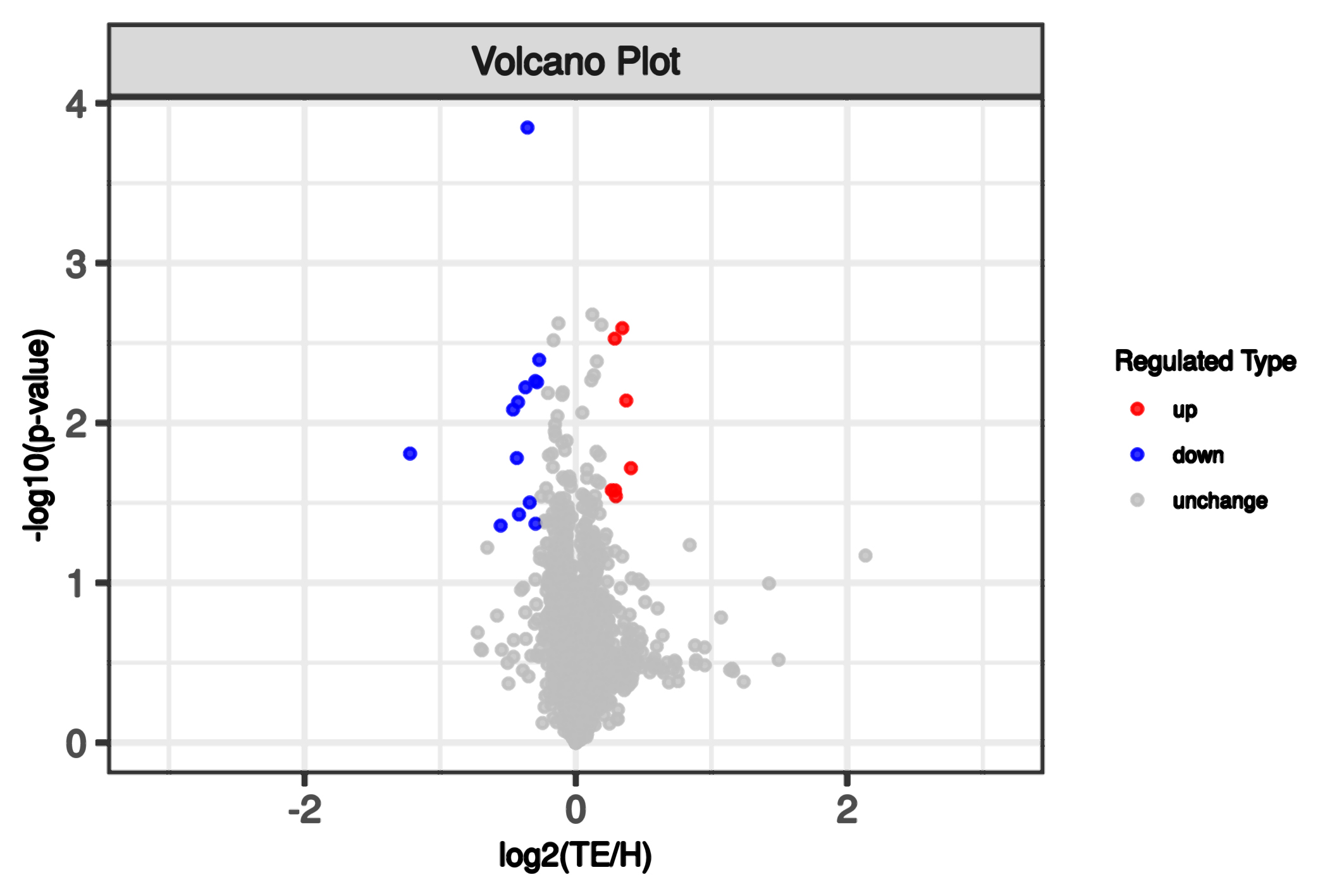

Supplement: Supplementary file 2 [file Image1.jpeg]

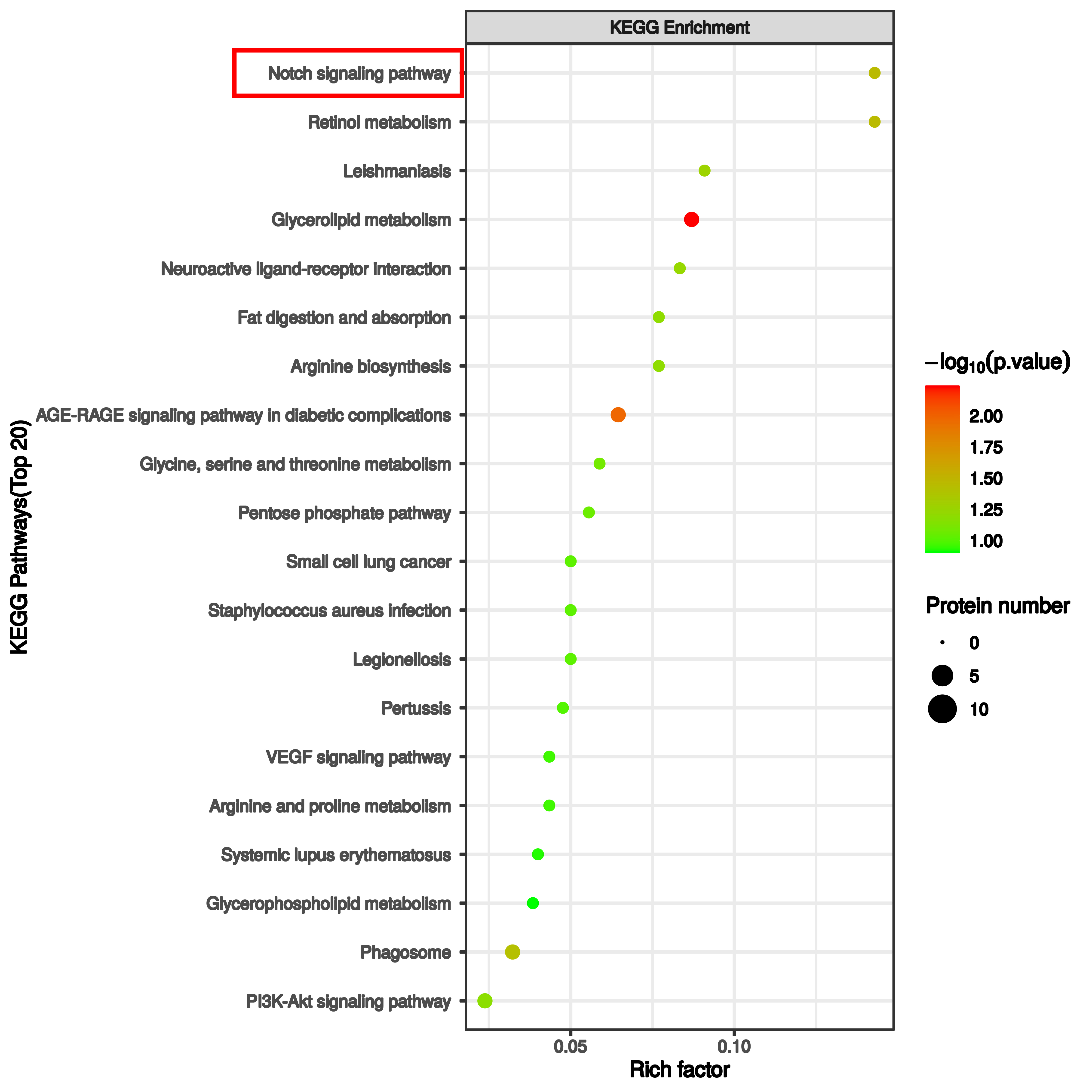

Supplement: Supplementary file 3 [file Image2.jpeg]

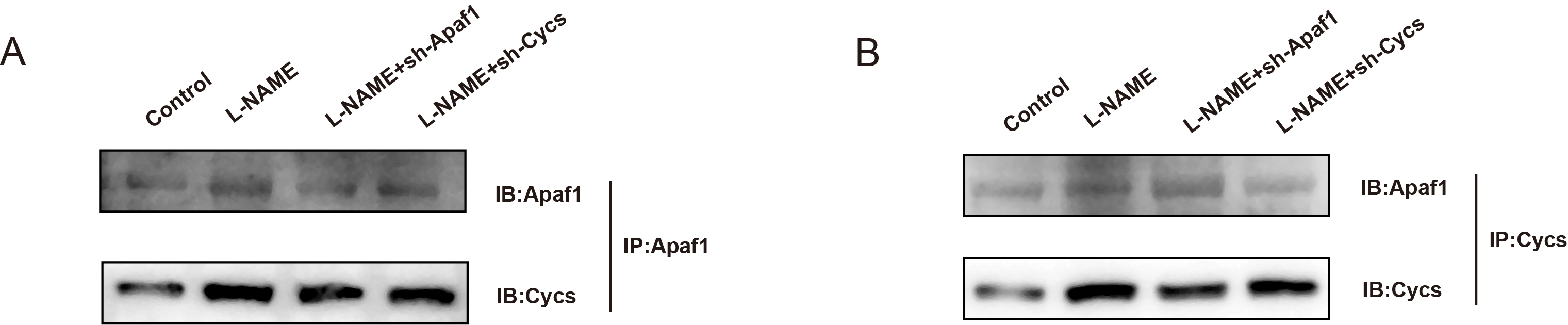

Supplement: Supplementary file 4 [file Image4.jpg]
